# Neurology, Neurorehabilitation and Aspects Related to Neuro-Quality of Life (Neuro-QoL)

**DOI:** 10.25122/jml-2019-1005

**Published:** 2019

**Authors:** Victor Lorin Purcarea

*“The 9th EUROPEAN TEACHING COURSE on NEUROREHABILITATION”, “The 14th INTERNATIONAL SUMMER SCHOOL OF NEUROLOGY”* and “*The 3rd TEACHING COURSE: TASK FORCE FOR RARE NEUROLOGIC DISEASES”* brought together a rich and diverse audience of healthcare professionals, researchers and leading academic scientists who shared, exchanged and valued experiences and results on all aspects, regarding neurorehabilitation, neurology and rare neurologic diseases in a variety of sessions.

**Figure 1:**
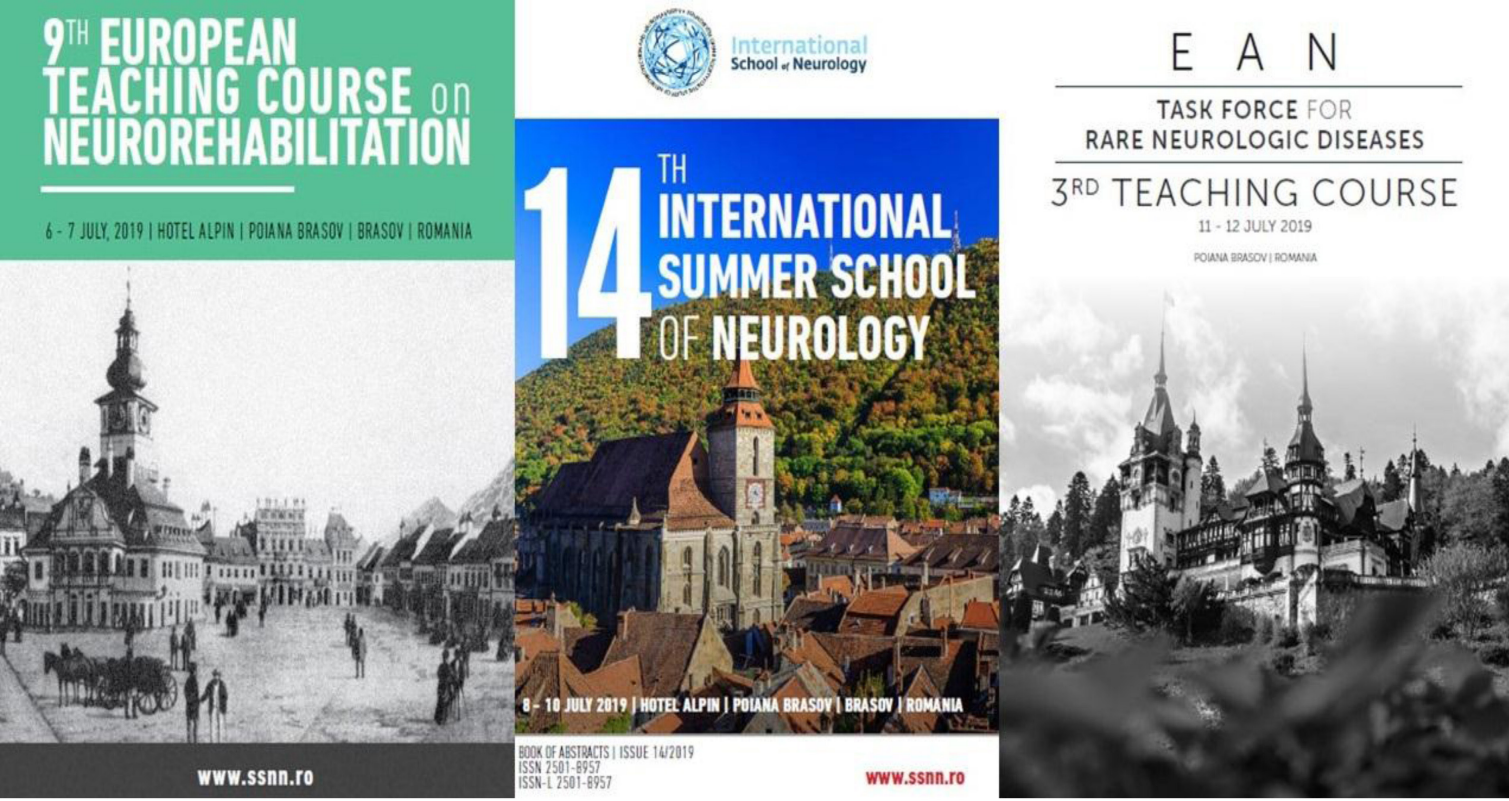
Posters of the events

The events organized by the **Foundation of the Society for the Study of Neuroprotection and Neuroplasticity (SSNN), Journal of Medicine and Life** being one of the academic partners, strived to advance the scientific and clinical knowledge and to address the latest issues regarding the best clinical practices, also training the next generation of healthcare professionals, providing cutting-edge lectures, while valuing mentorship.

The **Foundation of the Society for the Study of Neuroprotection and Neuroplasticity (SSNN)** has succeeded to establish an international academic framework during the past years, enabling international and local specialists to take part in open productive discussions.

SSNN organizes several international educational open stage events for debates annually, and its goal is to expand in the following years and set up an educational network available internationally.

Members of the presidium of *“The 9th EUROPEAN TEACHING COURSE on NEUROREHABILITATION”* from left to right: **DAFIN F. MUREȘAN** (was delivering the welcome speech at that moment) - EFNR President, Co-Chair EAN Scientific Panel Neurorehabilitation; **LEONARD SHEUNG WAI LI** (Hong Kong) - President of World Federation for NeuroRehabilitation; **DAVID C. GOOD** (USA) - Founding Chair of Neurology at the Milton S. Hershey Medical Center of Penn State College of Medicine; **VOLKER HÖMBERG** (GERMANY) - Program Chairman, EFNR Secretary General and WFNR Secretary General; **KARIN DISERENS** (Switzerland) - Acute Neurorehabilitation Unit, Neurology, Department of and Clinical Neurosciences, University Hospital of Lausanne; and **ALLA GUEKHT** (Russia) - Professor of Neurology, Russian National Research Medical University Director, Moscow Research and Clinical Center for Neuropsychiatry, Moscow.

**Figure 2:**
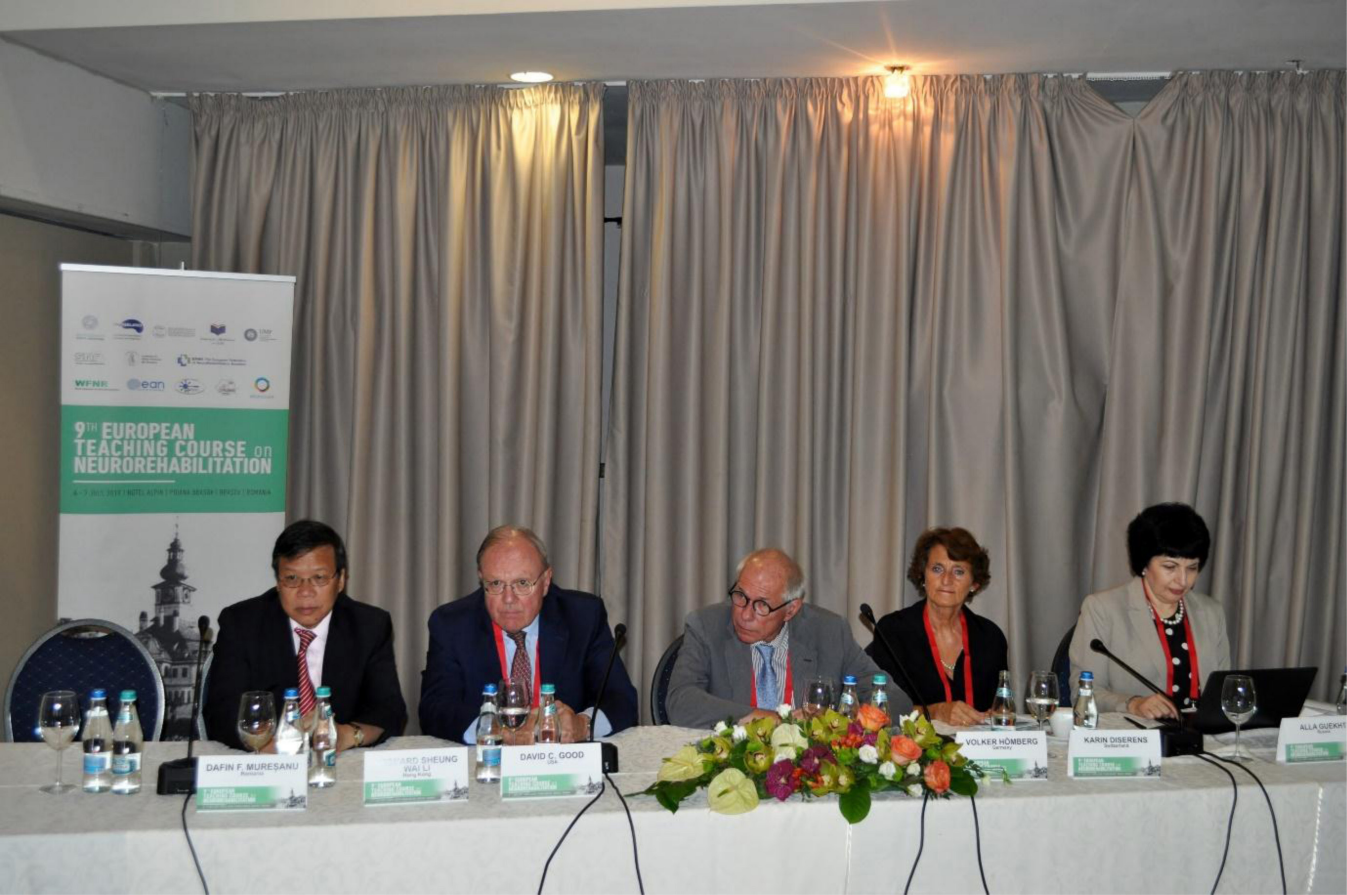
Members of the presidium of “The 9th EUROPEAN TEACHING COURSE on NEUROREHABILITATION”

**Figure 3:**
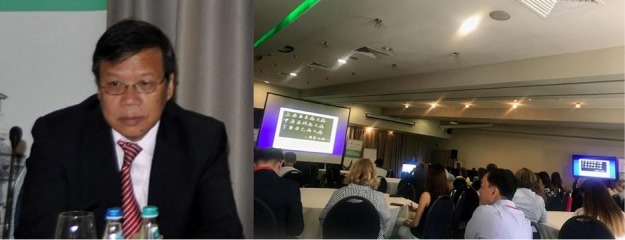
LEONARD SHEUNG WAI LI (Hong Kong) - President of World Federation for NeuroRehabilitation

**Leonard Sheung Wai Li** (Hong Kong) presented the topic “Diagnosis and management of hemiplegic shoulder pain”. He works in Hong Kong as Director of Neurological Rehabilitation Centre of Virtus Medical Group, taking position also as Honorary Clinical Professor of the Department of Medicine, University of Hong Kong and Adjunct Professor of the Department of Rehabilitation Sciences of Hong Kong Polytechnic University.

**DAFIN F. MUREȘAN** (Romania) presented the topic “Biomarkers of rehabilitation after stroke”. He is Professor of Neurology, Senior Neurologist, Chairman of the Neurosciences Department, Faculty of Medicine, “Iuliu Hatieganu” University of Medicine and Pharmacy Cluj-Napoca, President of the European Federation of Neurorehabilitation Societies (EFNR), Co-Chair EAN Scientific Panel Neurorehabilitation, Past President of the Romanian Society of Neurology, President of the Society for the Study of Neuroprotection and Neuroplasticity (SSNN), member of the Academy of Medical Sciences, Romania, secretary of its branch in Cluj and member of 17 scientific international societies (member of the American Neurological Association (ANA) and 10 national ones, being part of the executive board of most of these societies.

**Figure 4:**
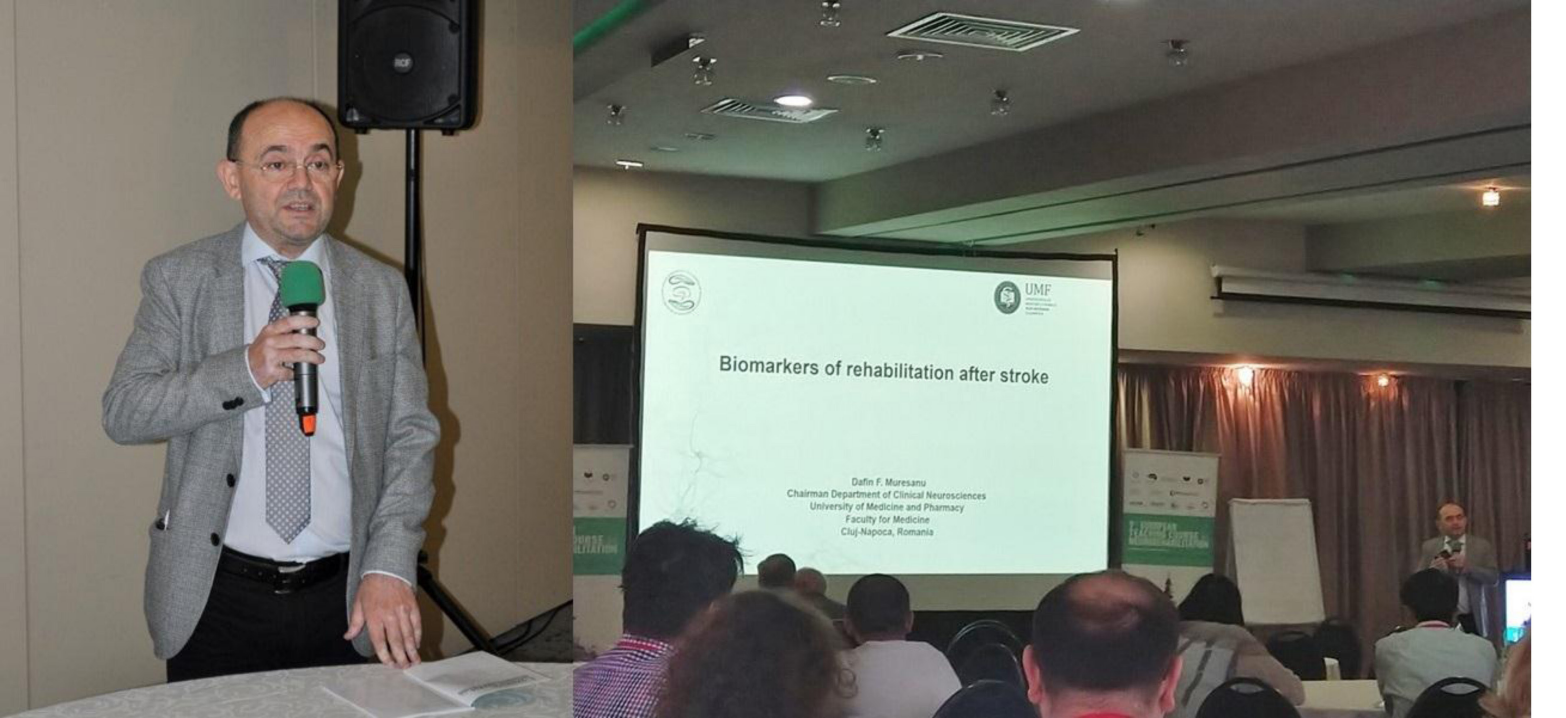
DAFIN F. MUREȘAN - EFNR President, Co-Chair EAN Scientific Panel Neurorehabilitation

**Figure 5:**
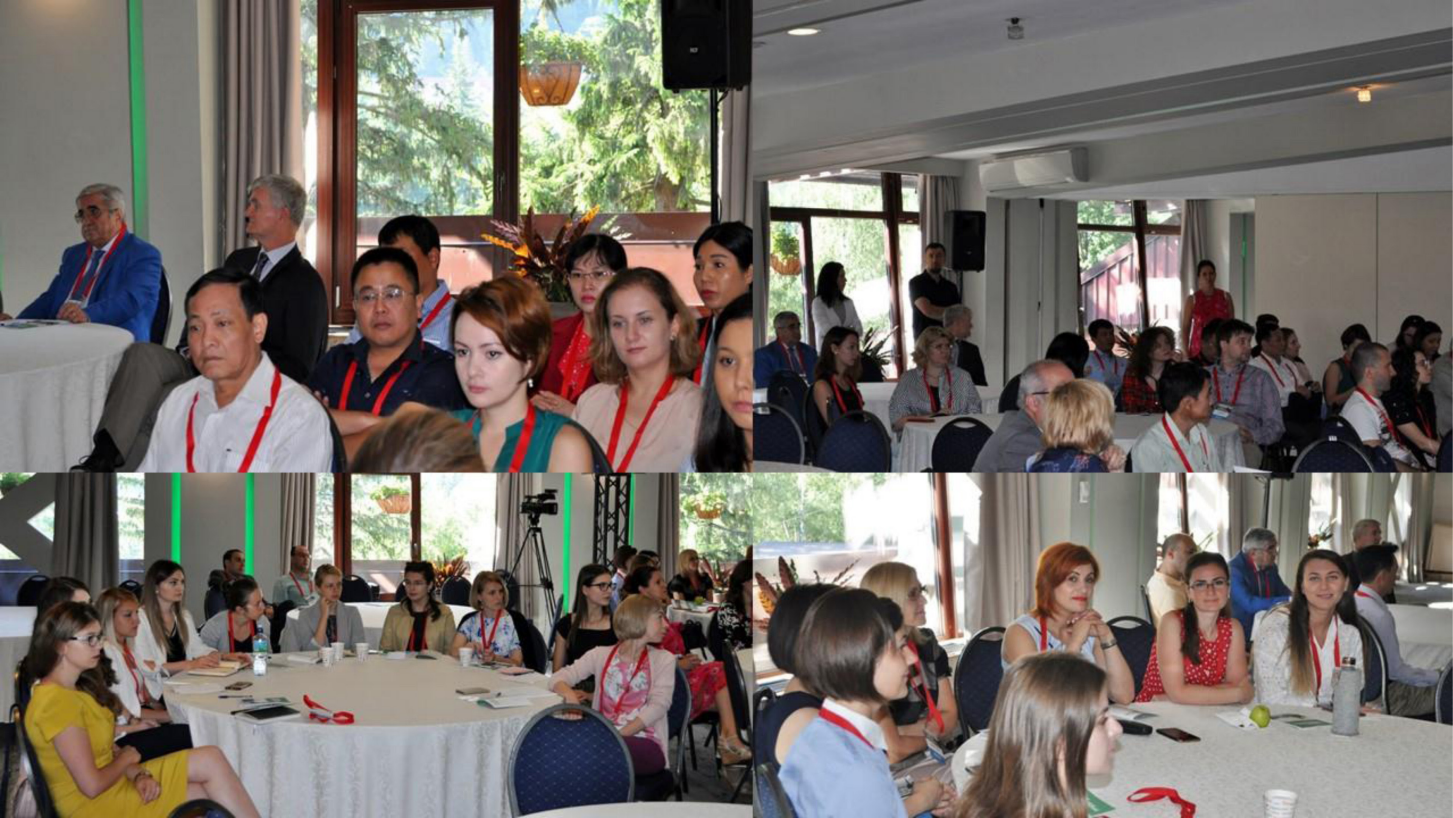
Audience of the event

The following events also offered the unique opportunity of sharing the latest breakthroughs with more connected interactions and experiences, building audience involvement, while discussing innovations and concerns adopted in Neurology.

“The human brain is by far the most complex physical object known to us in the entire cosmos” (Owen Gingerich - professor emeritus of astronomy and of the history of science at Harvard University and a senior astronomer emeritus at the Smithsonian Astrophysical Observatory)

While neurology is considered a practice of medicine dealing with disorders of the nervous system and with conditions, disease and, sometimes, rare diseases affecting the central and peripheral nervous systems (such as dementia, stroke, muscular dystrophy, stroke, neuropathy, Alzheimer, Parkinson and many others), neurorehabilitation, as a complex medical process, which has the aim of recovering after nervous system injuries, promotes the necessary skills to empower the patient to work at the highest level of independence, also encouraging to rebuild self-esteem.

In this context, neurorehabilitation should also be **patient-focused** with customized advanced healthcare strategies to bring the patient empowerment to another level; **participatory**, meaning that the patient and his family are actively involved during the treatment; and **community-focused**, when finding the best solutions adapted for the community reintegration is considered.

*Quality of Life* (QoL) represents the well-being of individuals and societies in general, highlighting the positive and negative aspects of life, in contrast to the *Health related quality of life* (HRQoL), which concentrates on the effects of illness and the impact that the treatment may have on Quality of Life. Thus, QoL is broader than HRQoL because of the inclusion of non-health related aspects of life, while HRQoL is directly connected to a persons’ status concerning its health or disease. In addition, the Neuro-Quality of life (Neuro-QoL), as a multidimensional patient-reported outcome measurement system, assesses conditions of mental, physical and social health that are identified, having also the ability to conduct comparisons for both PD-specific and cross-disease (Neuro-QoL health-related quality of life measurement system: Validation in Parkinson’s disease. Cindy J. Nowinski, Andrew Siderowf, Tanya Simuni, Catherine Wortman, Claudia Moy, David Cella Mov Disord. 2016 May; 31(5): 725–733. Published online 2016 Feb 26. doi: 10.1002/mds.26546 (PubMed). Neuro-QoL has been developed and validated for the most common neurological conditions and created for flexible administration, such as pen and paper, interview or web-based (HealthMeasures. http://www.healthmeasures.net/explore-measurement-systems/neuro-qol).

The instruments used in Neuro-QoL have been developed as a result of a collective research initiative sponsored by the **National Institute for Neurological Disorders and Stroke** in order to create clinically-relevant health-related quality of life and psychometrically-sound measurement tools for patients who have neurological conditions or disorders like amyotrophic lateral sclerosis (ALS), multiple sclerosis (MS), muscular dystrophy (MD), epilepsy, Parkinson’s disease (PD) or stroke.

From the beginning of the advancement and acceptance of Neuro-QoL measures, they have been enlarged for use in military deployment–related traumatic brain injury (MDR-TBI), traumatic brain injury (TBI), Huntington’s disease (HD) and spinal cord injury (SCI) (HealthMeasures. http://www.healthmeasures.net/explore-measurement-systems/neuro-qol).

Research concerning Neuro-QoL measures continues to spread, according to a 2016 study conducted by **Meehan and colleagues** that used Neuro-QoL measures to investigate whether the quality of life is affected at an older age due to sub-concussive blows during Division III college collision sports (Division III Collision Sports Are Not Associated with Neurobehavioral Quality of Life. J Neurotrauma. 2016 Jan 15;33(2):254-9. doi: 10.1089/neu.2015.3930. Epub 2015 Jul 20. (PubMed).

Research results acknowledged that “negative consequences of alcohol use” was the only outcome associated positively with the sub-concussive blows. Differently, participants with a concussion history, expressed worse self-reported health (such as executive function, cognition, anxiety, positive affect, sleep disturbance, depression, emotional and behavioral dyscontrol, negative consequences regarding alcohol use, fatigue).

The research highlighted that athletes having a concussion history might experience bad health outcomes at a later age, but the participation in collegiate level Division III collision sports (in itself) has no risk factors concerning bad long-term neurobehavioral results.

